# The agnarid terrestrial isopods (Isopoda, Oniscidea, Agnaridae) of the province of Qazvin, Iran, with a description of a new species

**DOI:** 10.3897/zookeys.515.9125

**Published:** 2015-07-30

**Authors:** Behjat Eshaghi, Bahram H. Kiabi, Ghasem M. Kashani

**Affiliations:** 1 Department of Zoology, Faculty of Biological Sciences, Shahid Beheshti University G.C. Tehran, Iran; 2 Department of Biology, Faculty of Science, University of Zanjan, Zanjan, Iran

**Keywords:** Oniscidea, Agnaridae, new species, Qazvin, Iran

## Abstract

Six species of terrestrial isopods from the province of Qazvin, central Iran, are recorded. Three species, *Hemilepistus klugii* (Brandt, 1833), *Protracheoniscus ehsani* Kashani, 2014 and *Mongoloniscus persicus* Kashani, 2014, were previously reported from the province. *Hemilepistus elongatus* Budde-Lund, 1885 and *Protracheoniscus major* (Dollfus, 1903) are recorded for the first time, and one species, *Protracheoniscus sarii* **sp. n.**, is described as new. The diagnostic characters of the new species are figured.

## Introduction

Several contributions on the terrestrial isopod fauna of Iran have recently been published (Khalaji-Pirbalouti and Wägele 2010; Kashani et al. 2010, 2011, 2013; Kashani and Sari 2012; Kashani 2014a, 2014b); however, the knowledge on this taxon remains relatively poor. During a survey of terrestrial isopods of the province of Qazvin, a dozen species of terrestrial isopods were collected. In the present study the species belonging to the family Agnaridae are investigated.

The family Agnaridae is characterized by possessing monospiracular covered lungs in all five pleopod-exopodites (Schmidt 2003, 2008). Distributed in the temperate and subtropical zones of Eurasia and northern Africa, the members of this family prefer habitats with low humidity (Schmidt 2003). According to the world catalogue of terrestrial isopods by Schmalfuss (2003), the family includes 15 genera, the validity of some of which is questionable (Ferrara and Taiti 1988). Up to date, three genera of Agnaridae, i.e. *Hemilepistus* Budde-Lund, 1879, *Protracheoniscus* Verhoeff, 1917 and *Mongloniscus* Verhoeff, 1930, were reported from Iran (Kashani et al. 2010; Kashani 2014a, 2014b) and three agnarid species, namely *Hemilepistus klugii* (Brandt, 1833), *Protracheoniscus ehsani* Kashani, 2014 and *Mongloniscus persicus* Kashani, 2014, were recorded from the province of Qazvin (Kashani et al. 2010; Kashani 2014b). Here, we report the occurrence of three more specie, one of which is new to science.

## Material and methods

The material of the present study was collected throughout the province of Qazvin. The specimens were collected by hand and preserved in 96% ethanol. Some of the specimens were dissected and the body parts were slide-mounted in Euparal (Carl Roth, Karlsruhe). Drawings were made using a camera lucida fitted on a SaIran ZSM-100 dissecting stereomicroscope and on a Nikon Y-IDT compound microscope. The specimens, including the type material of the newly described species have been deposited in the personal collection of the third author (PCGMK), the Zoological Museum, University of Tehran (ZUTC), and the Iranian Research Institute of Plant Protection, Tehran (IRIPP).

## Taxonomy

### Order Isopoda Latreille, 1817 Suborder Oniscidea Latreille, 1802 Family Agnaridae Schmidt, 2003

#### Genus *Hemilepistus* Budde-Lund, 1879

##### Hemilepistus klugii

Taxon classificationAnimaliaIsopodaAgnaridae

(Brandt, 1833)

###### Material examined.

Qazvin, 36°03.9'N, 50°03.6'E, 15 June 2008, leg. G.M. Kashani, two males and two females (ZUTC Iso.1059); Abgarm, Ardalan village, 35°53.6'N, 48°54.7'E, 21 June 2008, leg. G.M. Kashani, four males and six females (ZUTC Iso.1060); Boin-Zahra, Ebrahim-abad village, 10 October 2004, leg. M. Hakimzadeh, one male (PCGMK 1123); 5 km to Sagzabad, 35°46.4'N, 50°01.4'E, 18 June 2013, leg. G.M. Kashani & B. Eshaghi, one male (PCGMK 1656); Takestan to Zein-abad, 35°51.9'N, 49°52.5'E, 18 June 2013, leg. G.M. Kashani & B. Eshaghi, one male and two females (PCGMK 1660); Abgarm, 35°48.7'N, 49°08.0'E, 11 September 2013, leg. G.M. Kashani & B. Eshaghi, one male (PCGMK 1705a).

###### Remarks.

Kashani et al. (2010) reported the presence of *Hemilepistus klugii* in central parts of Iran, including the province of Qazvin. Here, more localities are presented for the species. This species occurs in semi-arid habitats of the province.

###### Distribution.

Azerbaidjan and central Iran.

##### Hemilepistus elongatus

Taxon classificationAnimaliaIsopodaAgnaridae

Budde-Lund, 1885

###### Material examined.

Takestan to Shal, 35°54.1'N, 49°48.0'E, 18 July 2013, leg. G.M. Kashani & B. Eshaghi, five males and two females (PCGMK 1661); Esfarvarin to Takestan, 35°58.0'N, 49°43.1'E, 18 July 2013, leg. G.M. Kashani & B. Eshaghi, one female (PCGMK 1664); Abyek to Gheshlagh, 36°01.3'N, 50°30.3'E, 10 September 2013, leg. G.M. Kashani & B. Eshaghi, one male and two females (PCGMK 1680).

###### Remarks.

Despite the broad distribution of *Hemilepistus elongatus* in Iran (Kashani and Sari 2012), this is the first time this species is reported from the province of Qazvin.

###### Distribution.

“Transcaucasus”; easternmost Turkey: Ararat; Turkmenia; Iran.

#### Genus *Mongoloniscus* Verhoeff, 1930

##### Mongoloniscus persicus

Taxon classificationAnimaliaIsopodaAgnaridae

Kashani, 2014

###### Material examined.

Boin Zahra, 30 June 2008, leg. G.M. Kashani, one male (PCGMK1627); Nikouieh, 36°16.2'N, 49°31.6'E, 11 September 2013, one male (PCGMK 1696); Abgarm, Chehel-Cheshmeh village, 35°46.6'N, 49°18.5'E, 11 September 2013, leg. G.M. Kashani & B. Eshaghi, four males and two females (PCGMK 1706); Qazvin to Nikouieh, 4 June 2014, leg. G.M. Kashani & B. Eshaghi, one female (PCGMK 1768); Nikouieh, Charandagh village, 4 June 2014, leg. G.M. Kashani & B. Eshaghi, three males and seven females (PCGMK 1770); Nikouieh to Manjil, 5 June 2014, leg. G.M. Kashani & B. Eshaghi, seven males and twelve females (PCGMK 1774b).

###### Remarks.

The presence of *Mongoloniscus persicus* in western Iran, including the province of Qazvin, was formerly reported by Kashani (2014b). Herein, only the sampling localities for the province are presented.

###### Distribution.

Western Iran.

#### Genus *Protracheoniscus* Verhoeff, 1917

##### Protracheoniscus major

Taxon classificationAnimaliaIsopodaAgnaridae

(Dollfus, 1903)

###### Material examined.

Saveh to Boin-Zahra, Seyd-abad village, 35°20.0'N, 50°13.0'E, 18 July 2013, leg. G.M. Kashani & B. Eshaghi, two males (PCGMK 1653); Boin-Zahra to Segzabad, 35°46.4'N, 40°03.0'E, 18 July 2013, leg. G.M. Kashani & B. Eshaghi, four females (PCGMK 1655b); Shal, 35°54.1'N, 49°48.0'E, 18 July 2013, leg. G.M. Kashani & B. Eshaghi, four males and three females (PCGMK 1662); Zia-abad, 36°00.6'N, 49°27.8'E, 18 July 2013, leg. G.M. Kashani & B. Eshaghi, one male and fifteen females (PCGMK 1667b); Takestan to Qazvin, Kahak village, 36°06.7'N, 49°45.0'E, 18 July 2013, leg. G.M. Kashani & B. Eshaghi, five females (PCGMK 1668b); Khakali, 36°08.2'N, 50°10.7'E, 10 September 2013, leg. G.M. Kashani & B. Eshaghi, two females (PCGMK 1686b); Koohin, 36°18.6'N, 49°48.8'E, 10 September 2013, leg. G.M. Kashani & B. Eshaghi, one female (PCGMK 1692a); Abhar, Darasajin village, 36°01.1'N, 49°14.2'E, 11 September 2013, leg. G.M. Kashani & B. Eshaghi, two males (PCGMK 1701b); Dowlat-abad to Abgarm, 35°55.5'N, 49°02.9'E, 10 September 2013, leg. G.M. Kashani & B. Eshaghi, one female (PCGMK 1704b); Abgarm, 35°48.6'N, 49°08.0'E, 10 September 2013, leg. G.M. Kashani & B. Eshaghi, two females (PCGMK 1705b); Takestan to Danesfahan, 35°52.5'N, 49°31.1'E, 12 September 2013, leg. G.M. Kashani & B. Eshaghi, one female (PCGMK 1713b); Qazvin to Nikouieh, Charandagh village, 4 June 2014, leg. G.M. Kashani & B. Eshaghi, one male (PCGMK 1764).

###### Remarks.

The presence of *Protracheoniscus major* in Iran was formerly reported by Kashani (2014a) but this is the first time it is recorded from the province of Qazvin. This species can be observed in high numbers especially in cultivated areas.

###### Distribution.

From middle Europe to Central Asia; Iran.

##### Protracheoniscus ehsani

Taxon classificationAnimaliaIsopodaAgnaridae

Kashani, 2014

###### Material examined.

Saveh to Boin-Zahra, Vardeh village, 35°15.2'N, 50°16.4'E, 18 July 2013, leg. G.M. Kashani & B. Eshaghi, eight males and five females (PCGMK 1652); Boin-Zahra to Sagzabad, 35°48.1'N, 49°52.5'E, 18 July 2013, leg. G.M. Kashani & B. Eshaghi, seven females (PCGMK 1658); Takestan to Shal, 35°54.1'N, 49°48.0'E, 18 July 2013, leg. G.M. Kashani & B. Eshaghi, one female (PCGMK 1663); Qazvin to Razmian, Barajin village, 19 July 2013, leg. G.M. Kashani & B. Eshaghi, eight females, two males and seven juveniles (PCGMK 1669); Qazvin to Razmian, Barajin village, 19 July 2013, leg. G.M. Kashani & B. Eshaghi, two females (IRIPP Iso.1048); 20 Km N Qazvin, 36°20.7'N, 50°10.7'E, 19 July 2013, leg. G.M. Kashani & B. Eshaghi, two males and seven females (PCGMK 1675); Khakali, 36°08.4'N, 50°10.7'E, 10 September 2013, leg. G.M. Kashani & B. Eshaghi, eight males and thirteen females (PCGMK 1685); 28 Km to Kouhin, 36°16.9'N, 49°56.9'E, 10 September 2013, leg. G.M. Kashani & B. Eshaghi, one female (PCGMK 1688); Nikouieh, 36°16.2'N, 49°31.7'E, 11 September 2013, leg. G.M. Kashani & B. Eshaghi, two females (PCGMK 1695); Abhar to Darasajin village, 36°03.7'N, 49°13.4'E, 11 September 2013, leg. G.M. Kashani & B. Eshaghi, three males and one female (PCGMK 1699); Darasajin village, 36°01.1'N, 49°14.3'E, 11 September 2013, leg. G.M. Kashani & B. Eshaghi, three males (PCGMK 1700); Dowlat-abad, 35°58.6'N, 49°08.6'E, 11 September 2013, leg. G.M. Kashani & B. Eshaghi, seven males and one female (PCGMK 1702); Dowlat-abad to Abgarm, Bouzandan village, 35°55.5'N, 49°02.9'E, 11 September 2013, leg. G.M. Kashani & B. Eshaghi, two males (PCGMK 1703); Abgarm to Takestan, Sagzenab village, 35°47.6'N, 49°22.8'E, 11 September 2013, leg. G.M. Kashani & B. Eshaghi, six males and sixteen females (PCGMK 1710); Takestan to Danesfahan, 35°52.5'N, 49°31.1'E, 11 September 2013, leg. G.M. Kashani & B. Eshaghi, two males and two females (PCGMK 1712); Nikouieh, Changooreh village, 4 June 2014, leg. G.M. Kashani & B. Eshaghi, one female (PCGMK 1767); Qazvin to Nikouieh, Changoureh village, 4 June 2014, leg. G.M. Kashani & B. Eshaghi, four males and six females (PCGMK 1769); 4 km to Nikouieh, 4 June 2014, leg. G.M. Kashani & B. Eshaghi, three males and eleven females (PCGMK 1771).

###### Remarks.

This species was recently described from central parts of Iran (Kashani, 2014b). Here more sampling localities for the province of Qazvin are provided.

###### Distribution.

Central Iran.

##### Protracheoniscus sarii sp. n.

Taxon classificationAnimaliaIsopodaAgnaridae

http://zoobank.org/334BDA58-C792-4808-8B47-F70AE0C55B75

Figures 1 , 2

###### Material examined.

Holotype: male, 7 mm, Qazvin, Khakali, 36°08.2'N, 50°10.6'E, 10 September 2013, leg. G.M. Kashani & B. Eshaghi, (ZUTC 5326).

**Paratypes.** Same data as holotype, one male (IRIPP Iso-1059); same data as holotype, one male (PCGMK 1684); Mali-Abad to Gheshlagh, 36°03.9'N, 50°19.7'E, 10 September 2013, leg. G.M. Kashani & B. Eshaghi, one female (IRIPP Iso-1055); Mali-Abad to Gheshlagh, 36°03.9'N, 50°19.7'E, 10 September 2013, leg. G.M. Kashani & B. Eshaghi, one male and two females (PCGMK 1682); 3 km to Avaj, 35°35.5'N, 49°13.3'E, 11 September 2013, leg. G.M. Kashani & B. Eshaghi, one male (PCGMK 1708).

###### Diagnosis.

Head with short lateral and developed rounded median lobes. Male pleopod exopodite I with a truncate apex; endopodite I apex bent outward, equipped with some small setae.

###### Description.

**Figure 1.**
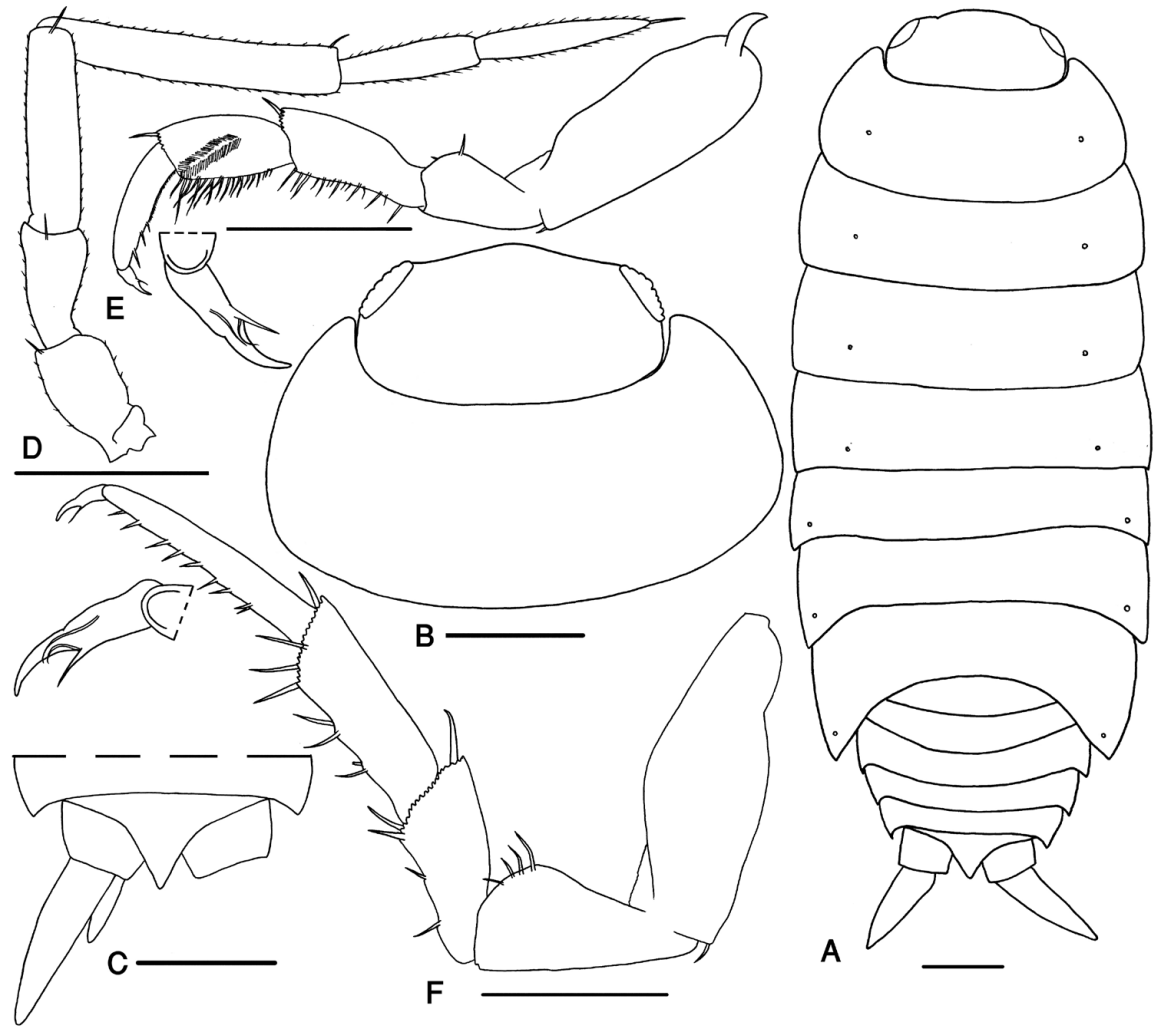
*Protracheoniscus sarii* sp. n., male, paratype. **A** body outline with position of noduli laterales **B** cephalon and first pereonite **C** telson and uropods **D** antenna **E** pereopod 1 **F** pereopod 7. Scale = 1 mm.

Maximum length of male and female 9 mm. Color brown with the usual pale muscle spots. Body outline as in Fig. 1A. Cephalon with rounded median lobe, protruding from the shorter lateral ones (Fig. 1B). Antenna long, surpassing the posterior margin of pereon tergite II; fifth article of peduncle as long as flagellum, with length: width ratio 6:1; flagellum with two articles, proximal article as long as the distal one (Fig. 1D). Pereopod I carpus with depression on rostral surface equipped with slender scales; propodus narrow and long, proximal part of sternal margin slightly concave with dense small scales, distal part bearing spine setae; pereopods I–VII dactylus with one dactylar and one ungual setae (Fig. 1E, F).

Pereon smooth. Pereon tergite I with rounded posterolateral margin. Noduli laterales on pereonites I to IV distinctly more distant from the lateral margins than those on pereonites V to VII (Fig. 1A).

Pleon narrower than pereon (Fig. 1A). Telson short, with distal part triangular bearing acute apex, slightly surpassing uropod protopodites (Fig. 1C). Uropod exopodites almost 1.5 times as long as telson (Fig. 1C). Pleopod exopodites I–V with monospiracular covered lungs (Fig. 1B–F).

**Figure 2.**
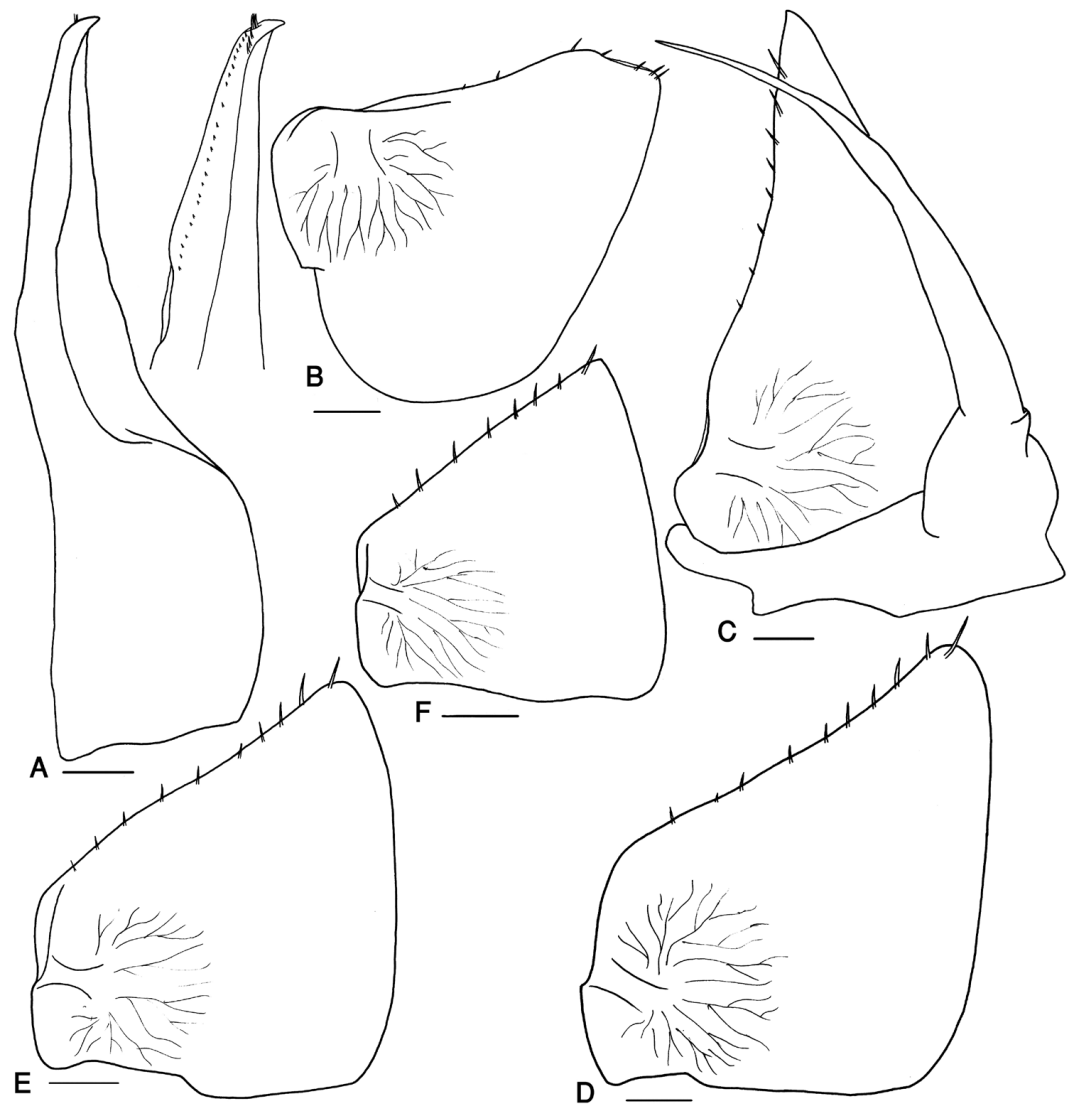
*Protracheoniscus sarii* sp. n., male, paratype. **A** pleopod endopodite I **B** pleopod exopodite I **C** pleopod II **D** pleopod exopodite III **E** pleopod exopodite IV **F** pleopod exopodite V. Scale = 0.1 mm

Male: Pereopods I–III merus and carpus with brushes of setae (Fig. 1E). Pereopod VII ischium triangular, with straight ventral margin, merus and carpus equipped with strong spines on sternal and distal margins (Fig. 1F). Pleopod exopodite I with long hind lobe and truncate distal margin (Fig. 2B); endopodite I straight with apical part triangular, bent outwards and equipped with small setae (Fig. 2A). Pleopod endopodite II slightly longer than exopodite; exopodite triangular with a line of strong setae on outer margin (Fig. 2C). Pleopod exopodites III–V as in Fig. 2D–F.

###### Etymology.

The name of the species is after Dr. Alireza Sari, professor in animal biosystematics, the University of Tehran, Iran.

###### Remarks.

*Protracheoniscus sarii* sp. n. is superficially similar to *Protracheoniscus ehsani* but differs in lacking the ridge on the dorsal margin of pereopod VII carpus, and the shape of pleopod endopodite I. Ecologically, this species is associated with relatively humid microhabitats.

###### Distribution.

Central Iran.

## Discussion

Several papers have been published on terrestrial isopod fauna of Iran, however, most parts of the country have not been properly investigated and certainly more taxa are present. Prior to this study, 35 species belonging to 21 genera and 11 families were reported from Iran. According to the present knowledge, two genera, *Brevurus* Schmalfuss, 1986 and *Pseudorthometopon* Schmalfuss, 1986, and thirteen species, *Psachonethes elbursanus* Schmalfuss, 1986, *Trachelipus azerbaidzhanus* Schmalfuss, 1986, *Trachelipus pieperi* Schmalfuss, 1986, *Cylisticoides rotundifrons* (Schmalfuss, 1986), *Hemilepistus schirasi* Lincoln, 1970, *Hemilepistus taftanicus* Kashani, Sari & Hosseinie, 2010, *Mongoloniscus persicus* Kashani, 2014, *Protracheoniscus gakalicus* Kashani, Malekhosseinie & Sadeghi, 2013, *Protracheoniscus ehsani* Kashani, 2014, *Brevurus masandaranus* Schmalfuss, 1986, *Porcellio rubidus* Budde-Lund, 1885 (nomen dubium), *Schizidium persicum* Schmalfuss, 1986 and *Pseudorthometopon martensi* Schmalfuss, 1986, are endemic to Iran.

## Supplementary Material

XML Treatment for Hemilepistus klugii

XML Treatment for Hemilepistus elongatus

XML Treatment for Mongoloniscus persicus

XML Treatment for Protracheoniscus major

XML Treatment for Protracheoniscus ehsani

XML Treatment for Protracheoniscus sarii
